# Machine Learning-Based Classification of Dependence in Ambulation in Stroke Patients Using Smartphone Video Data

**DOI:** 10.3390/jpm11111080

**Published:** 2021-10-25

**Authors:** Jong Taek Lee, Eunhee Park, Tae-Du Jung

**Affiliations:** 1 Artificial Intelligence Application Research Section, Electronics and Telecommunications Research Institute (ETRI), Daegu 42994, Korea; jongtaeklee@etri.re.kr; 2 Department of Rehabilitation Medicine, School of Medicine, Kyungpook National University, Daegu 41944, Korea; ehmdpark@knu.ac.kr; 3 Department of Rehabilitation Medicine, Kyungpook National University Chilgok Hospital, Daegu 41404, Korea

**Keywords:** machine learning, stroke, rehabilitation, dependent ambulation, postural balance

## Abstract

The goal of this study was to develop a framework to classify dependence in ambulation by employing a deep model in a 3D convolutional neural network (3D-CNN) using video data recorded by a smartphone during inpatient rehabilitation therapy in stroke patients. Among 2311 video clips, 1218 walk action cases were collected from 206 stroke patients receiving inpatient rehabilitation therapy (63.24 ± 14.36 years old). As ground truth, the dependence in ambulation was assessed and labeled using the functional ambulatory categories (FACs) and Berg balance scale (BBS). The dependent ambulation was defined as a FAC score less than 4 or a BBS score less than 45. We extracted patient-centered video and patient-centered pose of the target from the tracked target’s posture keypoint location information. Then, the extracted patient-centered video was input in the 3D-CNN, and the extracted patient-centered pose was used to measure swing time asymmetry. Finally, we evaluated the classification of dependence in ambulation using video data via fivefold cross-validation. When training the 3D-CNN based on FACs and BBS, the model performed with 86.3% accuracy, 87.4% precision, 94.0% recall, and 90.5% F1 score. When the 3D-CNN based on FACs and BBS was combined with swing time asymmetry, the model exhibited improved performance (88.7% accuracy, 89.1% precision, 95.7% recall, and 92.2% F1 score). The proposed framework for dependence in ambulation can be useful, as it alerts clinicians or caregivers when stroke patients with dependent ambulatory move alone without assistance. In addition, monitoring dependence in ambulation can facilitate the design of individualized rehabilitation strategies for stroke patients with impaired mobility and balance function.

## 1. Introduction

Stroke is the main cause of acquired disability in ambulation [1,2]. Impaired ambulation can be caused by motor weakness, sensory deficits, imbalance, visual impairment, or cognitive impairments following a stroke [3]. After rehabilitation, 64% of stroke survivors achieve independent ambulation, while 36% require assistance or are unable to walk [4]. Stroke survivors with impaired mobility and balance function are at higher risk for falls than healthy elderly individuals [5]. Most falls in stroke survivors occur while walking because the asymmetrical loadings between the paretic and non-paretic lower limbs impede balance control [6,7]. A recent cohort study found that dependence in ambulation is a predictive factor of functional independence and quality of life for stroke survivors [8].

Dependence in ambulation following a stroke is clinically evaluated by clinicians or physiotherapists relative to mobility and balance functions. Mobility function is commonly used to assess how much dependence or assistance is required. The functional ambulatory categories (FACs) are a common clinical assessment tool first described by Holden et al. [9]. FAC assessment provides information to stroke patients and caregivers about how much manual assistance by another person is required for walking [10]. Previous studies reported clinically frequent use of FACs in post-stroke assessment dependence in ambulation [10,11,12]. A FAC score of 4 or greater indicates community-dwelling ambulation at 6 months after a stroke [10], and a FAC score of less than 4 is a predicting risk factor of fear of falling at 12 months after stroke [12]. In addition, following a stroke, the balance function is evaluated using the Berg balance scale (BBS), which is an assessment tool initially developed to identify the risk of falls in the geriatric population [13]. The BBS provides information to caregivers about how to safely manage stroke patients [14]. The BBS scores for stroke patients have been shown to be strong predictors of the degree of dependence in ambulation [15,16]. A BBS score of less than 45 indicates the need for dependence or assistance in ambulation [15]. Therefore, determining independent or dependent ambulation using FAC and BBS scores is effective in terms of evaluating a functional ambulator in community return after stroke.

Human activity recognition, i.e., interpreting human body gestures or motions to determine human action, has received increasing attention in the field of computer vision over the past two decades [17,18]. Human activity recognition involves video-based human activity monitoring in various fields, e.g., healthcare [19], education [20], human–computer interaction [21], video surveillance [22], and sports [23,24]. In recent years, automated human activity recognition has been developed using machine learning and deep neural networks [25]. Among machine learning techniques, analyzing video using deep neural networks is a field that has received increasing attention [26,27,28]. According to a recent study, several human pose estimation frameworks efficiently extract and identify human joints from a given image of different people regardless of how many people are present in the image [29]. For example, a deep neural network has been developed to extract walking features, and this system performs well on untrained real-world data with high accuracy [30]. It is helpful for disabled patients to follow a rehabilitation strategy and monitor harmful situations in the community, e.g., the risk of falls [31]. However, to the best of our knowledge, no study has investigated the detection of dependent ambulation in a clinical setting. Thus, in this study, we used a deep neural network to classify dependence in ambulation in disabled stroke patients using video data acquired by a smartphone during inpatient rehabilitation therapy.

The purpose of this study was to determine dependence in ambulation of stroke patients using video data acquired by smartphone based on a 3D convolutional neural network (3D-CNN). Our primary contributions are summarized as follows:

First, the proposed framework can classify dependence in ambulation using video-recorded data using a smartphone in a natural situation. Second, to train our deep model on a small dataset, we applied feature extraction transfer learning from a trained model of Mobile Video Networks (MoViNet) and reduce intraclass variance by removing regions that are irrelevant to patients (e.g., the background). Third, to improve classification performance, we measured swing time asymmetry by analyzing pose keypoints and using them as supplementary results. Note that pose keypoints were already extracted to detect and track patients; thus, this process did not increase computation time.

## 2. Materials and Methods

**Figure 1:**
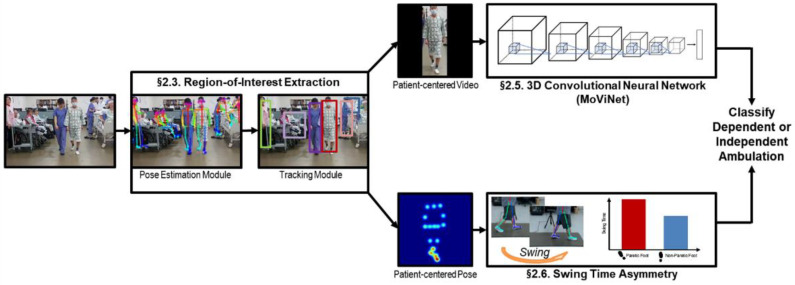
The design overview diagram for classification of dependence in ambulation in stroke patients.

The flowchart diagram of the overall system design is presented in Figure 1. The proposed system took video recordings from a smartphone as input. To extract the region of interest, the pose estimation module extracted keypoints of persons, and the tracking module tracks multiple persons simultaneously based on the locations of the keypoints (Section 2.3). Then, a clinician manually identified a stroke patient as a target from tracking multiple persons. The 3D-CNN took a patient-centered video of the target as input to classify the dependence in ambulation (Section 2.5). We measured the swing time asymmetry by analyzing step gait motion based on a patient-centered pose to improve classification performance in uncertain scores of dependence in ambulation (Section 2.6).

### 2.1. Video Data Collection

The video data were collected from 206 patients diagnosed with ischemic or hemorrhagic stroke who had received inpatient rehabilitation therapy at the Department of Rehabilitation Medicine at Kyungpook National University Chilgok Hospital from 7 January 2016 to 10 August 2019. In total, 351 videos were recorded by caregiver smartphones while patients received inpatient physical therapy (oral consent was provided by the patients). The videos were recorded in 960 × 540 and 640 × 360 resolutions and at 30 fps, and the length of each video ranged from 5.03 s to 135.8 s. The ambient light was fluorescent because the video was filmed indoors without windows. Here, patient faces were blurred to protect their identities. This retrospective study was approved by the Institutional Review Board at the Kyungpook National University Chilgok Hospital (No. KNUCH 2019-09-006).

### 2.2. Assessment of Dependence in Ambulation

The dependence in ambulation in stroke patients was evaluated as mobility and balance function by physiotherapists. Here, mobility function was assessed using the FAC score, in which a score of 0 indicates a patient that cannot walk at all or requires the help of two people; a score of 1 indicates a patient who requires continuous manual contact to support their body weight and maintain balance; a score of 2 indicates a patient who requires an intermittent or continuous light touch to assist balance or coordination; a score of 3 indicates a patient who can ambulate on a level surface without manual contact from another person but requires standby guarding against a person for either safety or verbal cueing; a FAC score of 4 indicates a patient who can ambulate independently on a level surface but requires supervision on stairs or uneven ground; lastly, a score of 5 indicates a patient who can walk independently in all environments, including stairs or uneven ground [9]. In this study, we defined dependence in ambulation following stroke as dependent ambulation with a FAC score of less than 4 and independent ambulation with a FAC score of 4 or greater [10,12].

In addition, balance function was assessed as the BBS score. The 14 items in the BBS are ordered according to increasing difficulty. The performance for each item is ranked on an ordinal scale from 0 to 4 with a maximum total score of 56 points. For each item, a score of 0 reflects the need for dependence or assistance to even minimally perform the requirements of the task, and a score of 4 reflects independence in maximal task performance [13]. A BBS score of less than 45 indicates the need for assistance or dependence during ambulation, and a BBS score of 45 or greater indicates independent ambulation [15].

### 2.3. Pose Estimation and Tracking for Region-of-Interest Extraction

We employed OpenPose, a real-time multi-person pose estimation library, to detect and track multiple persons [29]. Among tracked multiple persons, a clinician manually labeled a stroke patient as a target, the cropped videos of the target were used for classification of a dependent or independent ambulator based on video data acquired by a smartphone. 

First, the poses of all people in the video were estimated using a pose estimation framework for each frame in the video. Then, we applied the simple online and real-time tracking (SORT) method, which is a simple and efficient tracking method that is based on bounding boxes obtained from each person’s pose keypoints [32]. Here, to reduce person identity switch errors due to occluded objects, we adapted the object model using the representations of keypoint locations. Then, a clinician manually identified a stroke patient as a target from tracking multiple persons. We extracted patient-centered video and patient-centered pose of the target from the tracked target’s posture keypoint location information. Finally, the extracted patient-centered video was input in the 3D-CNN, and the extracted patient-centered pose was used to measure swing time asymmetry. Note that the proposed method is a pose-based detection and tracking method; thus, the soft image registration effect occurred.

### 2.4. Video Pre-Processing for Deep Learning

We split the videos acquired during inpatient rehabilitation therapy into multiple 5 s clips. As a result, we generated a total of 2311 clips. Here, 1218 cases (52.7%) involved the “walk” action, 690 cases involved the “stand” action, 260 cases involved the “sit” action, and 143 cases involved the “stair up” action. In further processing, we only used “walk” action cases because it represented the largest proportion of data with the most balanced independence/dependence during ambulation.

### 2.5. The 3D Convolutional Neural Network

Our solution provides a real-time determination of dependence in ambulation from video data of stroke patients walking. Therefore, MoViNet, which has demonstrated outstanding performance in terms of processing time and accuracy in a recently developed 3D-CNN, was used as the basic structure of our 3D-CNN [33].

MoViNet provides six sub-models (i.e., A0, A1, …, A5) according to image resolution and fps values. The A0 is the smallest model, and A5 is the largest model. We adapted a mid-size A2 model of the input of 224 × 224 pixels and 5 fps with the modification of input frame length from 10 s to 5 s. It was worth noting that the base model required 4.8 M parameters, and the amount of computation was 10.3 GFLOPS.

**Figure 2:**
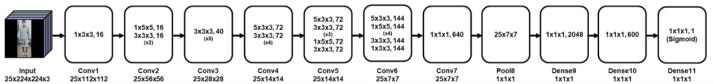
The 3D-CNN architecture for classification of dependence in ambulation (MoViNet-A2 structure). Conv, convolutional layer; Dense, dense layer; Pool, pooling layer.

The adapted model took as input a 4D tensor (25 × 128 × 128 × 3; time × width × height × color) constructed from patient-centered video segments with a uniform sampling rate of 5 Hz, which allowed us to optimize the size and quality of the video segments. Following the input layer, seven 3D convolutional blocks were connected in series. Each 3D convolutional block contained various combinations of 3D convolutional filters of 1 × 1 × 3, 1 × 3 × 3, 1 × 5 × 5, 3 × 3 × 3, 5 × 3 × 3, and 1 × 1 × 1 in series. After the convolutional blocks, a global averaging pooling layer summarized the feature maps over space and time. Then, three fully connected (dense) layers were used to output the binary classification decision of the dependence in ambulation of the patient in the video segment (Figure 2).

### 2.6. Swing Time Asymmetry Measurement

Stroke patients have asymmetry loadings between the paretic and non-paretic lower limbs while walking [7]. This feature is an important factor when determining dependence in ambulation. Thus, in this study, swing time asymmetry was used as an additional judgment basis of our framework. Swing time is defined as the time from the foot first leaving the ground (toe-off) to the time at which the same foot touches the ground (heel-strike). Here, the time was measured by tracking the position of the heel and toe keypoints of each foot in the patient-centered pose. To compute the time of heel strike and toe-off, we utilized the heel and toe keypoints, respectively, and determined the frame where the change in the sum of X-squared and Y-squared values was minimum across two consecutive frames.

**Figure 3:**
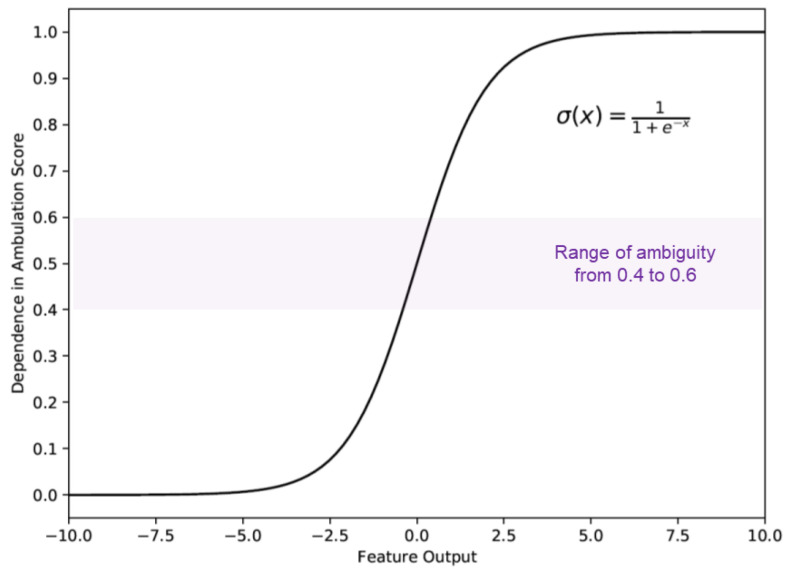
The optimized dependence in ambulation score of deep model output. Scores between 0.4 and 0.6 were considered uncertain results.

The output of the deep model (3D-CNN) had a value between 0 and 1 through a sigmoid function, as we trained our deep learning model with the sigmoid cross-entropy loss. The closer the value was to 1, the more dependent ambulation was, and the closer the value was to 0, the more independent ambulation was. However, a problem arose when this output was approximately 0.5, i.e., the uncertainty about the result was significant. In such cases, swing time asymmetry was measured, and the patient’s dependency was determined based on the measured swing time asymmetry value. In this study, we selected an optimal range value for deep model output ambiguity through experiments. When the range was 0.4 to 0.6, the accuracy rate was the lowest, and the re-decision by considering swing time asymmetry improved overall system accuracy (Figure 3). Swing time asymmetry is measured as follows:

$$\mathrm{Swing}\;\mathrm{time}\;\mathrm{asymmetry}=\frac{{\mathrm{Swing}\;\mathrm{time}}_{\mathrm{paretic}}}{{\mathrm{Swing}\;\mathrm{time}}_{\mathrm{non}-\mathrm{paretic}}}$$

When the patient’s swing time asymmetry value was close to 1.02, the system identified independent ambulation, and when the patient’s swing time asymmetry value was close to 1.24, the system identified dependent ambulation [7].

### 2.7. Training and Testing

To demonstrate the generalizability of the proposed model, we performed fivefold cross-validation for all experiments. Here, approximately 80% of the data were used as a training set, and the remaining data were used as the testing set. For example, the number of usable walking instances was 168; thus, the number of testing data was 34, and the number of the training data was 134. In addition, 25 consecutive frames were sampled uniformly from a long video sequence as input in the training phase. In the testing phase, the entire frames were used as input. The training module used stochastic gradient descent learning with cyclic learning rates [34]. The initial learning rate and the initial number of epochs were 5 × 10^−4^ and 10, respectively. The number of epochs was increased by a factor of two after each cycle. After performing three cycles, all models were trained for 70 epochs. Here, we employed binary cross-entropy as the loss function. The ground truth of the dependence in ambulation was confirmed from the FAC and BBS scores. If the FAC score was less than 4 or the BBS score was less than 45, the ground-truth label of the dependence in ambulation was positive (i.e., dependent ambulation); otherwise, the ground truth label was negative (i.e., independent ambulation).

### 2.8. Evaluation Metrics

To evaluate classification performance, accuracy, precision, recall, and F1 score values were measured. A correctly classified dependent patient and correctly classified independent patient were considered to be true positive (TP) and true negative (TN), respectively. A false positive (FP) was an independent patient classified incorrectly as a dependent patient, and a false negative (FN) was a dependent patient classified incorrectly as an independent patient.

The accuracy rate is the proportion of all correctly classified samples out of all samples, and it is defined by

$$\mathrm{Accuracy}=\frac{\mathrm{TP}+\mathrm{TN}}{\mathrm{TP}+\mathrm{TN}+\mathrm{FP}+\mathrm{FN}}$$

Precision is the proportion of TPs out of all samples predicted as dependent samples, and it is defined by

$$\mathrm{Precision}=\frac{\mathrm{TP}}{\mathrm{TP}+\mathrm{FP}}$$

Recall is the proportion of TPs out of all ground truth dependent patients, and it is defined by

$$\mathrm{Recall}=\frac{\mathrm{TP}}{\mathrm{TP}+\mathrm{FN}}$$

In addition, the F1 score is the harmonic mean of precision and recall, and it is defined by

$$F_{1}=\frac{2}{{\mathrm{Precision}}^{-1}+{\mathrm{Recall}}^{-1}}=\frac{2\cdot \mathrm{TP}}{2\cdot \mathrm{TP}+\mathrm{FP}+\mathrm{FN}}$$

We applied fivefold cross-validation; thus, we also present both the mean and standard deviation of each evaluation result.

## 3. Results

The demographic and clinical characteristics of 206 stroke patients who received inpatient rehabilitation therapy are shown in Table 1. The patients were 23 to 89 years old (mean age 63.24 ± 14.36 years; 108 males and 98 females). The number of ischemic stroke patients was 113 (54.9%), and the number of hemorrhagic stroke patients was 93 (45.1%). The time from stroke onset to video recording was 120.17 ± 281.52 days. During video recording, clinical assessments of dependence in ambulation were used as ground-truth labels when training the 3D-CNN framework. Based on the FAC score, the number of patients with dependent ambulation was 158 (76.7%), and the number of patients with independent ambulation was 48 (23.3%). Based on the BBS score, the number of patients with dependent ambulation was 152 (73.7%), and the number of patients with independent ambulation was 54 (26.3%).

**Figure 4:**
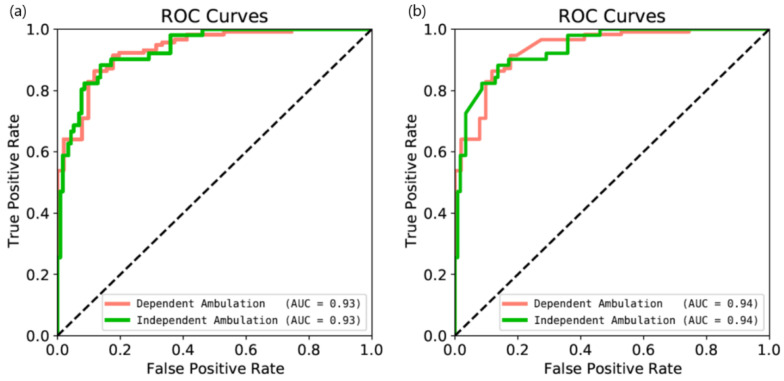
Receiver operating characteristic (ROC) curves for dependence in ambulation obtained: (**a**) using only the 3D-CNN and (**b**) when combined with measurement swing time asymmetry. The green line indicates independent ambulation following stroke and the pink line indicates dependent ambulation following stroke. AUC, the area under the curve.

Table 2 shows the detection performance using the 3D-CNN according to whether dependence in ambulation was determined using either FAC, BBS, or both. When training the 3D-CNN based on the FAC score, the model obtained 84.5% accuracy, 85.3% precision, 92.8% recall, and 88.8% F1 score. When training the 3D-CNN based on the BBS score, the model obtained 85.1% accuracy, 86.3% precision, 91.6% recall, and 88.6% F1 score. In addition, when training the 3D-CNN based on both the FAC and BBS scores, the model shows improved performance (86.3% accuracy, 87.4% precision, 94.0% recall, and 90.5% F1 score). The area under the curve (AUC) was 0.93 for dependent ambulation and 0.93 for independent ambulation, as shown in Figure 4a.

To improve detection performance, we extracted and calculated swing time asymmetry in the patient-centered module. Then, the result from the 3D-CNN was combined with the swing time asymmetry values, as shown in Table 3. When the 3D-CNN based on both the FAC and BBS values was combined with swing time asymmetry, the model improved performance (88.7% accuracy, 89.1% precision, 95.7% recall, and 92.2% F1 score). Here, the AUC curve was 0.94 for dependent ambulation and 0.94 for independent ambulation, as shown in Figure 4b.

## 4. Discussion

We proposed a deep learning framework for the classification of dependence in ambulation using video data acquired by a smartphone during inpatient rehabilitation therapy for stroke patients. The proposed framework demonstrated a high detection accuracy for both dependent and independent ambulation via transfer learning of a state-of-the-art 3D-CNN and efficient combination of swing time asymmetry analysis. The results of this study provide information that we expect to be valuable in fall prevention when stroke patients with dependent ambulation attempt to move independently. To the best of our knowledge, no previous study has investigated the use of machine learning analysis to determine dependence in ambulation in stroke patients using video data acquired by a smartphone.

There was a high correlation between the FAC and BBS scores; thus, the F1 score demonstrated good performance at 83% and 86%, respectively, when analyzed using each score. However, we found that the FAC and BBS scores were not completely consistent; thus, we combined the FAC and BBS scores, and we obtained an F1 score of 90%, which was an improvement of approximately 2%, compared when using each score independently.

We found that clinical assessments, e.g., the FAC and BBS scores, help measure a stroke patient’s ability to walk; however, there are two main problems to address. First, clinical assessments should be performed by trained clinicians or physiotherapists with sufficient time. Second, even if the FAC and BBS scores are measured, identifying a patient found in CCTV to retrieve clinical assessment scores is not permitted in many countries due to privacy issues. Our proposed framework can estimate dependence in ambulation from video data without extracting personal information. Furthermore, most studies on falls were collected retrospectively through questionnaires. This retrospective data collection did not fully reflect the risk of falls or fall incidence in the community. Given the high incidence of falls in elderly and stroke survivors, classifying dependence in ambulation in our framework can be important to prevent falls.

In research settings, machine learning techniques have been used in qualitative analyses during walking, thus modeling biomechanical systems by determination of the relationship between input data and outputs [35]. The input data were primarily collected using a motion capture system and electromyography, including kinematics, kinetics, or neuromuscular signals from the trunk and lower limb movements during walking [36,37]. Recent machine learning studies have analyzed various sensor data from infrared cameras, accelerometers, inertial measurement units, and pressure as input data [38,39,40,41]. Although qualitative data were not included in this study, we also proposed a method to measure swing time asymmetry during walking in real time using video trained using a pose estimation module. It can be used to quickly measure asymmetric temporal parameters when walking using only video data without various sensor data.

In addition, several limitations need to be addressed. First, we did not apply image pre-processing; however, performing a denoising technique can improve the system’s performance if the images are noisy [42]. Second, as only swing time asymmetry was analyzed, we did not investigate other spatiotemporal parameters during walking, e.g., step length and velocity. In the future, we aim to estimate various spatiotemporal parameters during walking using 3D pose estimation. Third, we only analyzed video data; however, it may be beneficial to also analyze audio data because smartphones record both audio and video, and audio data can be robust to occlusion. Finally, we extracted the rectangular bounding box of the persons, but the segmentation of images related to the regions of interest can provide relevant information on the posture of the patients [43,44].

## 5. Conclusions

In this study, we proposed a deep learning framework that can classify the dependence in ambulation in stroke patients with high performance. The trained 3D-CNN performed with 86.3% accuracy, 87.4% precision, 94.0% recall, and 90.5% F1 score. The trained 3D-CNN combined with measuring swing time asymmetry improved performance in 88.7% accuracy, 89.1% precision, 95.7% recall, and 92.2% F1 score. The proposed framework can be easily used in hospitals or local communities because it uses video captured by a smartphone. This system can alert medical staff and caregivers in real time when a stroke patient with dependent ambulation moves alone without any assistance or supervision. These warnings will help prevent falls in stroke patients. Furthermore, monitoring ambulation using videos may facilitate the design of personalized rehabilitation strategies for stroke patients with ambulatory and balance deficits in the community.

## Figures and Tables

**Table 1:** Demographic and clinical characteristics of stroke patients.

Parameters	Patients (*n* = 206)
Age (mean ± SD, years)	63.24 ± 14.36
Sex (male: female, *n*)	108:98
Stroke (ischemic: hemorrhagic, *n*)	113:93
Involved stroke lesion	
Right: left:both hemisphere (*n*)	82:105:19
Supratentorial: infratentorial lesion (*n*)	156:50
Vascular territory in ischemic stroke (*n* = 113) (ACA:MCA:PCA:BA/SCA/PICA/AICA/VA, *n*)	0:81:3:29
Classification of hemorrhagic stroke (*n* = 93) (ICH:IVH:SAH:SDH, *n*)	69:1:18:5
Time from stroke onset to recorded video (mean ± SD, days)	120.17 ± 281.52
Level of dependence in ambulation when recording video	
FAC score (mean ± SD)	1.73 ± 1.82
FAC < 4 (dependent):FAC ≥ 4 (independent) (*n*, %)	158 (76.7):48 (23.3)
BBS score (mean ± SD)	23.75 ± 20.56
BBS < 45 (dependent): BBS ≥ 45 (independent) (*n*, %)	152 (73.7):54 (26.3)

ACA, anterior cerebral artery; AICA, anterior inferior cerebellar artery; BA, basilar artery; BBS, Berg balance scale; FAC, functional ambulatory category; ICH, intracerebral hemorrhage; IVH, intraventricular hemorrhage; MCA, middle cerebral artery; SAH, subarachnoid hemorrhage; SCA, superior cerebellar artery; SDH, subdural hemorrhage; PCA, posterior cerebral artery; PICA, posterior–inferior cerebellar artery; VA, vertebral artery.

**Table 2:** The detection scores (accuracy, recall, precision, and F-1 score) of dependence in ambulation using a trained 3D-CNN model.

3D-CNN When Training Based on Assessment Scores	Accuracy	Precision	Recall	F-1 Score
FAC	0.845 ± 0.065	0.853 ± 0.057	0.928 ± 0.055	0.888 ± 0.050
BBS	0.851 ± 0.037	0.863 ± 0.065	0.916 ± 0.046	0.886 ± 0.032
FAC and BBS	0.863 ± 0.032	0.874 ± 0.024	0.940 ± 0.035	0.905 ± 0.022

BBS, Berg balance scale; FAC, functional ambulatory category.

**Table 3:** The detection scores (accuracy, recall, precision, and F-1 Score) of dependence in ambulation using a trained 3D-CNN model combined with measurement swing time asymmetry.

**3D-CNN with Swing Time Asymmetry**	**Accuracy**	**Precision**	**Recall**	**F-1 Score**
	0.887 ± 0.044	0.891 ± 0.041	0.957 ± 0.028	0.922 ± 0.029

## Data Availability

Data sharing is not applicable.

## References

[1] Langhorne P., Bernhardt J., Kwakkel G. (2011). Stroke rehabilitation. Lancet.

[2] Perry J., Garrett M., Gronley J.K., Mulroy S.J. (1995). Classification of walking handicap in the stroke population. Stroke.

[3] Mohan D.M., Khandoker A.H., Wasti S.A., Ismail Ibrahim Ismail Alali S., Jelinek H.F., Khalaf K. (2021). Assessment Methods of Post-stroke Gait: A Scoping Review of Technology-Driven Approaches to Gait Characterization and Analysis. Front. Neurol..

[4] Jørgensen H.S., Nakayama H., Raaschou H.O., Olsen T.S. (1995). Recovery of walking function in stroke patients: The Copenhagen Stroke Study. Arch. Phys. Med. Rehabil..

[5] Xu T., Clemson L., O'Loughlin K., Lannin N.A., Dean C., Koh G. (2018). Risk factors for falls in community stroke survivors: A systematic review and meta-analysis. Arch. Phys. Med. Rehabil..

[6] Forster A., Young J. (1995). Incidence and consequences offalls due to stroke: A systematic inquiry. Bmj.

[7] Patterson K.K., Gage W.H., Brooks D., Black S.E., McIlroy W.E. (2010). Evaluation of gait symmetry after stroke: A comparison of current methods and recommendations for standardization. Gait Posture.

[8] Chang W.H., Sohn M.K., Lee J., Kim D.Y., Lee S.-G., Shin Y.-I., Oh G.-J., Lee Y.-S., Joo M.C., Han E.Y. (2016). Predictors of functional level and quality of life at 6 months after a first-ever stroke: The KOSCO study. J. Neurol..

[9] Holden M.K., Gill K.M., Magliozzi M.R., Nathan J., Piehl-Baker L. (1984). Clinical gait assessment in the neurologically impaired: Reliability and meaningfulness. Phys. Ther..

[10] Mehrholz J., Wagner K., Rutte K., Meiβner D., Pohl M. (2007). Predictive validity and responsiveness of the functional ambulation category in hemiparetic patients after stroke. Arch. Phys. Med. Rehabil..

[11] van Bloemendaal M., van de Water A.T., van de Port I.G. (2012). Walking tests for stroke survivors: A systematic review of their measurement properties. Disabil. Rehabil..

[12] Goh H.-T., Nadarajah M., Hamzah N.B., Varadan P., Tan M.P. (2016). Falls and fear of falling after stroke: A case-control study. PMR.

[13] Berg K.O., Maki B.E., Williams J.I., Holliday P.J., Wood-Dauphinee S.L. (1992). Clinical and laboratory measures of postural balance in an elderly population. Arch. Phys. Med. Rehabil..

[14] Blum L., Korner-Bitensky N. (2008). Usefulness of the Berg Balance Scale in stroke rehabilitation: A systematic review. Phys. Ther..

[15] Berg K., Wood-Dauphinee S., Williams J. (1995). The Balance Scale: Reliability assessment with elderly residents and patients with an acute stroke. Scand. J. Rehabil. Med..

[16] Louie D.R., Eng J.J. (2018). Berg Balance Scale score at admission can predict walking suitable for community ambulation at discharge from inpatient stroke rehabilitation. J. Rehabil. Med..

[17] Popoola O.P., Wang K. (2012). Video-based abnormal human behavior recognition—A review. IEEE Trans. Syst. Man Cybern. Part C (Appl. Rev.).

[18] Ann O.C., Theng L.B. Human activity recognition: A review. Proceedings of the 2014 IEEE International Conference on Control System, Computing and Engineering (ICCSCE 2014).

[19] Arifoglu D., Bouchachia A. (2017). Activity recognition and abnormal behaviour detection with recurrent neural networks. Procedia Comput. Sci..

[20] Chintalapati S., Raghunadh M. Automated attendance management system based on face recognition algorithms. Proceedings of the 2013 IEEE International Conference on Computational Intelligence and Computing Research.

[21] Sharma R.P., Verma G.K. (2015). Human computer interaction using hand gesture. Procedia Comput. Sci..

[22] Wang L., Qiao Y., Tang X. Action recognition with trajectory-pooled deep-convolutional descriptors. Proceedings of the IEEE Conference on Computer Vision and Pattern Recognition.

[23] Pareek P., Thakkar A. (2021). A survey on video-based human action recognition: Recent updates, datasets, challenges, and applications. Artif. Intell. Rev..

[24] Thomas G., Gade R., Moeslund T.B., Carr P., Hilton A. (2017). Computer vision for sports: Current applications and research topics. Comput. Vis. Image Underst..

[25] Wu D., Sharma N., Blumenstein M. Recent advances in video-based human action recognition using deep learning: A review. Proceedings of the 2017 International Joint Conference on Neural Networks (IJCNN).

[26] Ji S., Xu W., Yang M., Yu K. (2012). 3D convolutional neural networks for human action recognition. IEEE Trans. Pattern Anal. Mach. Intell..

[27] Tran D., Bourdev L., Fergus R., Torresani L., Paluri M. Learning spatiotemporal features with 3d convolutional networks. Proceedings of the IEEE International Conference on Computer Vision.

[28] Carreira J., Zisserman A. Quo vadis, action recognition? a new model and the kinetics dataset. Proceedings of the IEEE Conference on Computer Vision and Pattern Recognition.

[29] Cao Z., Hidalgo G., Simon T., Wei S.-E., Sheikh Y. (2019). OpenPose: Realtime multi-person 2D pose estimation using Part Affinity Fields. IEEE Trans. Pattern Anal. Mach. Intell..

[30] Alharthi A.S., Yunas S.U., Ozanyan K.B. (2019). Deep learning for monitoring of human gait: A review. IEEE Sens. J..

[31] Ke S.-R., Thuc H.L.U., Lee Y.-J., Hwang J.-N., Yoo J.-H., Choi K.-H. (2013). A review on video-based human activity recognition. Computers.

[32] Bewley A., Ge Z., Ott L., Ramos F., Upcroft B. Simple online and realtime tracking. Proceedings of the 2016 IEEE International Conference on Image Processing (ICIP).

[33] Kondratyuk D., Yuan L., Li Y., Zhang L., Tan M., Brown M., Gong B. Movinets: Mobile video networks for efficient video recognition. Proceedings of the IEEE/CVF Conference on Computer Vision and Pattern Recognition.

[34] Smith L.N. Cyclical learning rates for training neural networks. Proceedings of the 2017 IEEE Winter Conference on Applications of Computer Vision (WACV).

[35] Khera P., Kumar N. (2020). Role of machine learning in gait analysis: A review. J. Med Eng. Technol..

[36] Van Gestel L., De Laet T., Di Lello E., Bruyninckx H., Molenaers G., Van Campenhout A., Aertbeliën E., Schwartz M., Wambacq H., De Cock P. (2011). Probabilistic gait classification in children with cerebral palsy: A Bayesian approach. Res. Dev. Disabil..

[37] Yoo T.K., Kim S.K., Choi S.B., Kim D.Y., Kim D.W. Interpretation of movement during stair ascent for predicting severity and prognosis of knee osteoarthritis in elderly women using support vector machine. Proceedings of the 2013 35th Annual International Conference of the IEEE Engineering in Medicine and Biology Society (EMBC).

[38] Paulo J., Peixoto P., Amorim P. (2019). Trajectory-based gait pattern shift detection for assistive robotics applications. Intell. Serv. Robot..

[39] Cho J.-s., Cho Y.-S., Moon S.-B., Kim M.-J., Lee H.D., Lee S.Y., Ji Y.-H., Park Y.-S., Han C.-S., Jang S.-H. (2018). Scoliosis screening through a machine learning based gait analysis test. Int. J. Precis. Eng. Manuf..

[40] Guo G., Guffey K., Chen W., Pergami P. (2017). Classification of normal and pathological gait in young children based on foot pressure data. Neuroinformatics.

[41] Kashi S., Polak R.F., Lerner B., Rokach L., Levy-Tzedek S. (2020). A machine-learning model for automatic detection of movement compensations in stroke patients. IEEE Trans. Emerg. Top. Comput..

[42] Ouahabi A. A review of wavelet denoising in medical imaging. Proceedings of the 2013 8th International Workshop on Systems, Signal Processing and Their Applications (WoSSPA).

[43] Ouahabi A., Taleb-Ahmed A. (2021). Deep learning for real-time semantic segmentation: Application in ultrasound imaging. Pattern Recognit. Lett..

[44] Arbaoui A., Ouahabi A., Jacques S., Hamiane M. (2021). Concrete Cracks Detection and Monitoring Using Deep Learning-Based Multiresolution Analysis. Electronics.

